# Fertility Histories and Heart Disease in Later Life in China

**DOI:** 10.3389/fpubh.2022.819196

**Published:** 2022-06-02

**Authors:** Yuanyang Wu, Jiahui Pang, Jiahao Wang, Jing Wu, Shuo Zhang, Siqing Zhang, Yidan Yao, Simeng Cheng, Yiwen Tao, Zheng Shen, Zhi-yun Li, Lin Xie, Hualei Yang

**Affiliations:** ^1^School of Public Administration, Zhongnan University of Economics and Law, Wuhan, China; ^2^School of Economics and Management, Zhejiang Agriculture and Forestry University, Zhejiang, China; ^3^College of Politics and Public Administration, Qingdao University, Qingdao, China; ^4^Chinese Academy of Social Sciences (CASS), Beijing, China

**Keywords:** fertility, heart disease, life course, the elderly, CHARLS

## Abstract

**Purpose:**

Based on life course theories, health among older people is driven by a continuous and cumulative process that develops over the life course. To better understand the aging process, it is important to assess associations between parity and heart disease in older people of China.

**Method:**

The associations between heart disease prevalence and number of births, number of boys or girls ever born were evaluated among 5,990 samples (mean age 64.1 years) using the Probit regression model based on the data from China Health and Retirement Longitudinal Study (CHARLS) conducted in 2013 and 2018. The model was adjusted only for rural or urban residents, and multivariate regression models were run separately by gender.

**Results:**

Our results showed that more than three children or more than two boys ever born were associated with a higher risk of heart disease. However, the number of girls ever born had no significant effect on heart disease in the elderly. We further analyzed the group difference between urban and rural residents using the regression model. More than three children or more than two boys ever born were associated with a high risk of heart disease in rural areas. Compared to urban residents, rural residents were more likely to be suffering from heart disease due to high parity. When considering the digender difference the paper found that more than three children ever born were associated with a high risk of heart disease in the female group. Late age at the time of giving birth for the first time was associated with a poorer risk level of heart disease in the rural residents, because the phenomenon of early childbearing was serious in the rural residents. But after considering the impact on the physical health of using chronic diseases, the first birth and the last birth both increased the risk of heart disease.

**Conclusions:**

Some policy implications were being put forward. Firstly, parents who were ready to give birth should be aware of the possible health loss of high parity. Postpartum nutrition supplements and chronic disease prevention were suggested to prevent heart disease in later life. Secondly, the elderly in rural areas should pay more attention to heart diseases. Participating in more daily exercise and physical examinations was a good choice to reduce the risk of heart disease. Thirdly, women who give birth prematurely have a higher risk of CVD. Based on our results, age at entry to parenthood was closely related to the risk of heart disease in later life.

## Introduction

It is well known that life expectancy in China has increased for decades, and this trend is likely to continue, thanks to medical improvement. Over the same period of time, China Longitudinal Aging Social Survey (CLASS) conducted in 2014 reported that 75.2% of the urban and rural elderly suffered from chronic diseases. Health inequalities among older people have become starker. Consequently, heightened morbidity and longer periods spent with a lower quality of life have become serious threats for larger segments of the older population. It is important to focus on these threats and identify major risk factors to better understand the aging process.

Several life course theories hypothesize that health in adulthood is the result of early life conditions and even critical moments in the early period (1, 2), and health among older people is driven by a continuous and cumulative process that develops over the life course (3). According to these points, health at a specific point in time is determined by all of the events that had happened previously. Therefore, it is important to take into account key events across the life courses to fully understand health conditions among older people. Among these events, the topic of fertility history about health has been widely discussed. The association between parity and physical and mental health has been found in recent studies (4–6). However, most of the previous studies have focused less on older people in China, whose elderly population aged 60 and above has reached 264 million, accounting for 18.7% of the total population^1^. As a typical representative of an aging country, it is necessary to consider the long-term effects of fertility history in older people in China.

The life course theory originated from the Chicago School in the United States in the 1920s. Life span, life history, and life cycle are the sources of life course theory. The discovery of transition and role change in Life span, the time concept of age research in life history, and the inspiration of relationship and role in the life cycle, three analytical traditions have created a three-dimensional life course theory composed of three concepts: trajectory, turning point, and opportunity. Life-course can be defined as “a series of socially defined events and roles played by individuals over time,” and it contained four core principles in the paradigm of life course research: space and time, opportunity, social connection, and individual initiative. The widespread concern of life course theory in social sciences began in the 1960s. Life-course theory opened a new perspective to explore life and health, that is, important events that occurred in the life course of individuals at different times. With the change of space and the passage of time, the influence of individual health trajectories on important events showed a dynamic accumulation process. Because the life course theory pays attention to the time effect of the environment and events, it can effectively combine the changes in Chinese social history with the health of the elderly. Therefore, the life course theory provides strong theoretical support for the cumulative impact of fertility events on the health of the elderly, and it is of great significance to explore the changing trend of healthy aging.

As previous studies have shown based on the life course theory, most studies have demonstrated the negative effects of high parity on health in later life. As far as the relationship between the number of children born to women and cardiovascular disease (CVD) is concerned, women whose children number is over 5 had worse cardiovascular health (7) and were more likely to suffer from CVD (8), women without children had a lower CVD prevalence (9). According to the Framingham Heart Study, compared with women who had never been pregnant, women with a history of more than 5 pregnancies had a significantly higher risk of developing CVD (10). Other studies pointed out that there may be a J-shaped relationship between the number of female children and CVD. Women with two children were at the lowest point of developing CVD, while women with more children were more likely to develop various types of CVD (11, 12), including coronary heart disease (CHD) (13, 14), ischemic heart disease (15), stroke (16, 17), and carotid atherosclerosis (18). The number of children and CVD mortality was U-shaped (19). However, after adjusting for other factors, there was uncertainty about whether this positive correlation was still significant or not. Parikh et al.'s (11) study showed that after accounting for the socioeconomic factors and the pregnancy-related complications, the conclusion of the J-shaped relationship between parity and CVD was still significant. The study of Parikh et al. eliminated the relationship between female ischemic heart disease mortality and parity after adjusting for education level and marital status. Nonetheless, there were also studies that conflicted with the above conclusions. In a study of 119,963 American women, researchers found that a number of children, parity, and the age of the first birth was not significantly associated with CVD (20). In the Rancho Bernardo Study, a high number of pregnancies would reduce the mortality of women with CVD, non-coronary heart disease, and non-CVD.

In addition to parity, age at entry to parenthood was also associated with the risk of heart diseases. Early pregnancy was positively related to the possibility of women suffering from CVD. A study of 7,600 British men and women found that giving birth before the age of 20 could cause women to be associated with an adverse cardiovascular profile in their middle age. Similarly, parents who give birth at a later age had a lower vascular risk (21). In a similar study of 3,937 Finnish women, the researchers found that compared with those who gave birth at the age of 31–35, women who have completed their first childbirth at or before 25 are more likely to develop myocardial infarction and arrhythmia (22). Women's age at first childbirth was positively correlated with hypertension and high cholesterol (23). On the contrary, Parikh et al. (24) found that women's age at first childbirth was negatively correlated with coronary heart disease (CHD).

Overall, most of the previous studies have focused less on older people in China and it is worth considering whether the existing association applies to China or not. Using the data from China Health and Retirement Longitudinal Study (CHARLS) conducted in 2013 and 2018, this study aimed to explore the association between parity and the risk of heart disease for the elderly in China. Furthermore, we looked at differences in this association across different gender and regions and compared results of boys and girls ever born respectively.

## Method

### Sample

We used data from the China Health and Retirement Longitudinal Study (CHARLS) conducted in 2013 and 2018, a nationally representative longitudinal study of the older population of China. This data covers 28 provinces, 150 counties (districts), and 10,624 families. Considering the needs of the study, people aged 60 and above were selected as the research objects. After eliminating the missing values of key variables, 5,990 observations were finally reserved. Table 1 showed the number of respondents with complete data for each variable used in the analysis.

**Table 1:** Descriptive analysis.

	**Men**			**Women**		
**varname**	**obs**	**Mean**	**Std**.	**obs**	**Mean**	**Std**.
heart_disease	2,718	0.197	0.398	3,272	0.244	0.429
childnum	2,718	2.993	1.616	3,272	3.131	1.733
age	2,718	64.785	10.741	3,272	63.817	11.061
age^2^	2,718	4312.500	1426.033	3,272	4194.934	1437.872
marriage	2,718	0.752	0.431	3,272	0.594	0.491
hukou	2,718	0.222	0.416	3,272	0.217	0.412
edu	2,718	5.948	3.848	3,272	3.863	3.927
livechild	2,718	0.534	0.498	3,272	0.557	0.496
disabled	2,718	0.078	0.269	3,272	0.097	0.296
finance	2,718	515.4	3.651	3,272	276.7	3.572

### Measures

The explained variable in this paper is a dummy variable related to heart disease. In the questionnaire of CHARLS (China Health and Retirement Longitudinal Study) in 2013 and 2018, respondents were asked that “*Have you been diagnosed with Heart attack, coronary heart disease, angina, congestive heart failure, or other heart problems by a doctor?”*. If the respondents were suffering from heart disease, their answers would be recorded and assigned a value of 1. Otherwise, the answer would be assigned a value of 0. So, the explained variable was a dummy variable used to measure the risk of heart disease in the elderly.

There were three explanatory variables, including the number of children (variable name: childnum), number of boys (variable name: boynum) ever born and the number of girls ever born (variable name: girlnum). We calculated the number of children per respondent through the “*Child data,”* a part of the overall data. Considering the influence of Chinese family structure, the role boys and girls played in Chinese families was significantly different. It was necessary to analyze the effects of boys and girls on heart disease in the elderly. The number of children, number of boys, and number of girls were measured using continuous variables indicating the number of children the elderly had.

### Covariates

We used personal characteristics, family information, and financial status to set covariates. Age was measured in single years. Considering the non-linear relationship, the square term of age (variable name: age^2^) was also included. Gender was measured using a dummy variable, female was 1 and male was 0. The marital status was divided into two categories, marriage or widowed, divorce (variable name: marriage). The respondents from rural areas or urban areas were also considered in the model (variable name: hukou). There was an obvious difference between rural and urban residents. Educational attainment was measured by the length of education he or she had received (variable name: edu). Living arrangements were divided into living with children and not living with children (variable name: livechild). We also considered the disabled person (variable name: disabled). If the respondent was disabled, the answer would be assigned a value of 1. Otherwise, the answer would be assigned a value of 0. We used financial assets to measure the impact of economic status (variable name: finance). More financial assets mean higher economic status.

### Analysis

Because of the previous findings in the literature showing a differing impact of childbearing on men and women, the descriptive analysis was performed separately by gender. We used the Probit model to examine the association between the risk of heart disease for the elderly and number of children. Multivariate regression models were run to estimate the association between the different fertility variables and the risk of heart disease.

First, to have a general picture of the associations, an analysis was performed on all the samples. The risk of heart disease was looked at by running three Probit models, each one focusing on a different fertility characteristic: parity, the number of boys ever born, and the number of girls ever born. Each specification included the control variables, including personal characteristics, family information, and financial status. Then, the marginal effect of each model was looked at.

Secondly, because of the literature showing a differing impact of childbearing on gender and region, the following multivariate regression models were run separately by gender and region. Comparing the results across different gender and region allowed an evaluation of whether the results were influenced by differences in the group or not.

This paper examined the effect of fertility on health through ten regression models. Each model focused on the age at the first birth or last birth and analyzed the group difference. The analyses were performed using STATA, reporting different levels of statistical significance of the coefficients (^*^p < 0.10, ^**^p < 0.05, ^***^p < 0.01).

## Result

### Descriptive Analysis of the Sample

The sample included 2,718 men and 3,272 women. Of these, 19.7% of men were suffering from heart disease and 24.4% of women had heart disease, with a significant mean difference. 79.3% of all respondents had four children or less, the maximum of children being 11. The average age of respondents was 64.1, the age distribution was between 60 and 99. Generally, age was associated with the linear and quadratic slopes, so the square of age was considered in the model. The marital status was divided into two categories, marriage or widowed and divorce. 47.1% of respondents were in the marriage category. Fewer than 30% were urban residents while rural residents were the majority of the sample. 77.4% of respondents had received education for 6 years or less, which was equivalent to the level of primary school, indicating that the level of education of respondents was a little low. 8.83% of respondents were disabled, having difficulties in eating, showering, or other daily activities. The log of financial assets was between 0 and 2,957,929 yuan. Through descriptive analysis, we could initially understand the relevant sample information.

### Regression and Group Difference

In Table 2, the (1)-(3) columns are the OLS regression results, and the (4)-(6) columns are the Probit regression results. If the number of children is > 3, then the risk of heart disease is higher. If the number of boys is > 2, then the risk of heart disease is higher. Associations between the number of children and heart disease showed that high parity and the number of boys ever born were associated with a higher risk of heart disease. However, the number of girls ever born had no significant effect on the heart disease of the elderly. Other covariates were also associated with the heart disease of the elderly. There was a non-linear relationship between age and risk of heart disease. The inflection point of age was 72.5, indicating that the older the elderly, the higher risk of heart disease before 72.5. After 72.5, the risk declined. Elderly women were associated with a higher risk of heart disease than men, which was consistent with descriptive analysis. These control variables (“hukou,” “edu,” “disabled”) were associated with a high risk of heart disease. And these control variables (“marriage,” “livechild,” “finance”) were slightly associated with the risk of heart disease.

**Table 2:** The regression results of the number of children and heart disease.

	**OLS**			**Probit**		
	**(1)**	**(2)**	**(3)**	**(4)**	**(5)**	**(6)**
(0= less than 3)						
childnum>3	0.040^***^			0.145^***^		
	(0.015)			(0.051)		
(0=less than 2)						
boynum>2		0.027^*^			0.103^**^	
		(0.015)			(0.052)	
girlnum>2			0.009			0.031
			(0.014)			(0.051)
age	0.014^***^	0.027^***^	0.027^***^	0.055^***^	0.100^***^	0.098^***^
	(0.005)	(0.006)	(0.006)	(0.020)	(0.021)	(0.021)
age^2^	−0.000^**^	−0.000^***^	−0.000^***^	−0.000^**^	−0.001^***^	−0.001^***^
	(0.000)	(0.000)	(0.000)	(0.000)	(0.000)	(0.000)
gender	0.077^***^	0.071^***^	0.071^***^	0.274^***^	0.254^***^	0.255^***^
	(0.011)	(0.011)	(0.011)	(0.041)	(0.041)	(0.041)
marriage	0.017	0.006	0.005	0.062	0.023	0.019
	(0.014)	(0.014)	(0.014)	(0.050)	(0.049)	(0.049)
hukou	0.108^***^	0.112^***^	0.111^***^	0.352^***^	0.370^***^	0.365^***^
	(0.015)	(0.015)	(0.015)	(0.047)	(0.047)	(0.047)
edu	0.008^***^	0.007^***^	0.007^***^	0.027^***^	0.025^***^	0.025^***^
	(0.002)	(0.002)	(0.002)	(0.006)	(0.006)	(0.006)
livechild	−0.011	−0.011	−0.010	−0.045	−0.045	−0.042
	(0.010)	(0.010)	(0.010)	(0.038)	(0.038)	(0.038)
disabled	0.129^***^	0.126^***^	0.126^***^	0.414^***^	0.407^***^	0.408^***^
	(0.022)	(0.022)	(0.022)	(0.063)	(0.063)	(0.063)
finance	0.002	0.002	0.002	0.007	0.008	0.007
	(0.002)	(0.002)	(0.002)	(0.006)	(0.006)	(0.006)
time fixe effect	YES	YES	YES	YES	YES	YES
_cons	−0.484^***^	−0.971^***^	−0.961^***^	−3.436^***^	−5.139^***^	−5.099^***^
	(0.172)	(0.187)	(0.187)	(0.661)	(0.716)	(0.715)
*N*	5,990	5,990	5,990	5,990	5,990	5,990
*R* ^2^	0.044	0.052	0.051			

* *p < 0.10*,

** *p < 0.05*,

*** *p < 0.001*.

The particularity of China lies in the dual structure of urban and rural areas. There are obvious differences in the level of economic development between urban and rural areas. Considering the difference, we further analyzed the group difference of urban-rural using the regression. In Table 3, high parity and more boys ever born were associated high risk of heart disease in rural areas. Compared to urban residents, rural residents were more likely to be suffering from heart disease due to high parity. Better medical and living conditions may mediate the negative effects of high parity in urban areas.

**Table 3:** The group difference in urban-rural.

	**Rural (Probit)**			**Urban (Probit)**		
	**(7)**	**(8)**	**(9)**	**(10)**	**(11)**	**(12)**
(0=less than 3)						
childnum>3	0.103^*^			−0.138		
	(0.060)			(0.117)		
(0=less than 2)						
boynum>2		0.153^***^			−0.001	
		(0.059)			(0.112)	
girlnum>2			0.049			−0.027
			(0.058)			(0.109)
control	YES	YES	YES	YES	YES	YES
variables						
_cons	−4.690^***^	−4.780^***^	−4.751^***^	−5.546^***^	−5.658^***^	−5.647^***^
	(0.823)	(0.824)	(0.823)	(1.462)	(1.465)	(1.460)
*N*	4,575	4,575	4,575	1,415	1,415	1,415

* *p < 0.10*,

^**^p < 0.05,

*** *p < 0.001*.

Table 4 also showed the association between the number of children and heart disease when considering the gender difference. Models stratified by gender indicated that more children ever born were associated with a high risk of heart disease in women, which was consistent with the results of Parikh et al. (24). There was no evidence showing that the number of boys and girls ever born were associated with high heart disease when stratifying the analysis by gender.

**Table 4:** The group difference in gender.

	**Men (Probit)**			**Women (Probit)**		
	**(13)**	**(14)**	**(15)**	**(16)**	**(17)**	**(18)**
(0=less than 3)						
childnum>3	−0.061			0.116^*^		
	(0.087)			(0.068)		
(0=less than 2)						
boynum>2		0.126			0.086	
		(0.078)			(0.069)	
girlnum>2			0.011			0.051
			(0.079)			(0.067)
control variables	YES	YES	YES	YES	YES	YES
_cons	−7.033^***^	−7.029^***^	−7.011^***^	−3.961^***^	−4.000^***^	−3.962^***^
	(1.251)	(1.254)	(1.254)	(0.886)	(0.887)	(0.885)
*N*	2,718	2,718	2,718	3,272	3,272	3,272

* *p < 0.10*,

^**^p < 0.05,

*** *p < 0.001*.

### The Association Between Early or Late Parenthood and Heart Disease

In addition to parity, age at entry to parenthood was also associated with the risk of heart disease in the elderly. Late age at entry to parenthood was associated with a lower risk level of heart disease. This association remained significant after adjusting for rural residents only.

In Table 5, the result showed that the age at which a person had their last child had a weak effect on their risk of heart disease. From the perspective of statistics, there seemed to be no obvious correlation between late age at last birth and the risk of heart disease for the elderly. The relationship between the two needed to be explored further based on more comprehensive samples.

**Table 5:** The regression results between the age of born and heart disease.

	**Rural**		**Urban**	
	**(19)**	**(20)**	**(21)**	**(22)**
firstborn	−0.019^***^	−0.011		
	(0.006)	(0.010)		
lastborn			−0.008	−0.011
			(0.005)	(0.008)
Control variables	YES	YES	YES	YES
_cons	−4.277^***^	−5.307^***^	−4.363^***^	−5.125^***^
	(0.836)	(1.482)	(0.846)	(1.508)
*N*	4,575	1,415	4,575	1,415

^*^p < 0.10,

^**^p < 0.05,

*** *p < 0.001*.

In Table 6, the paper used the chronic disease as a moderating variable and add it to the model. We found that the coefficient of chronic disease is significantly positive (0.013). The conclusion showed that those in better health were less likely to develop heart disease in old age. Therefore, physical health is an important factor in the relationship between the age to give birth and the risk of heart disease. Therefore, when in good health, the negative effect of the fertility behavior can be restrained.

**Table 6:** The moderating effect of chronic disease.

	**Rural**		**Urban**	
	**(23)**	**(24)**	**(25)**	**(26)**
firstborn	−0.042^***^	−0.035^***^		
	(0.007)	(0.010)		
Firstborn^*^disease	0.016^***^	0.013^***^		
	(0.001)	(0.001)		
lastborn			−0.025^***^	−0.029^***^
			(0.005)	(0.009)
Lastborn^*^disease			0.012^***^	0.011^***^
			(0.001)	(0.001)
Control variables	YES	YES	YES	YES
_cons	−4.616^***^	−5.169^***^	−4.673^***^	−5.023^***^
	(0.871)	(1.519)	(0.881)	(1.569)
*N*	4,575	1,415	4,575	1,415

* *p < 0.10*,

^**^p < 0.05,

*** *p < 0.001*.

## Discussion

In contemporary aging populations, the health of the elderly presents one of the greatest challenges faced by individuals, families, and society. Our results show associations between the number of births and the risk of heart disease. Although a considerable amount of covariance was explained by socioeconomic factors, some of the associations remained even after adjusting for several factors related to health, social contacts, and isolation. The association was consistent with the existing conclusions in foreign samples. As far as the relationship between the number of children born to women and CVD is concerned, women with many children (≥5) have worse cardiovascular health (7) and are more likely to suffer from CVD (8), women without children have a lower CVD prevalence (9). According to the Framingham Heart Study, compared with women who have never been pregnant, women with a history of more than 5 pregnancies have a significantly higher risk of developing CVD (10). Other studies pointed out that there may be a J-shaped relationship between the number of female children and CVD. Women with two children are at the lowest point of developing CVD while women with more children are more likely to develop various types of CVD (11, 12), including coronary heart disease (CHD) (13–15), stroke (16, 17), and carotid atherosclerosis (18).

In the case of high parity, the high risk of heart disease was affected by the factors combined with physiological factors. The trade-off hypothesis between fertility and longevity supported the negative effect on health brought by multiple births. High parity would bring malnutrition, physical loss, and other disadvantages to health. Meanwhile, the metabolic processes occurring during pregnancy such as changes in lipids, glucose, and weight may partly explain the increased burden of cardiovascular disease (CVD) among multi-parous women later in life. The accumulation of health disadvantages became the inducement of chronic disease, including heart disease. At the same time, social factors were considered to partly explain the formation of the relationship. On the one hand, children may exert social control on behavior and provide emotional and practical support, with potentially beneficial implications for the parents' health behavior. On the other hand, there may also be less favorable effects. In particular, earnings may be reduced as a result of childrearing, while expenses are high, and the parents may be under intense time pressure. This may lead to less healthy food intake and reduce the parents' physical activity (25).

When adjusting the model only for rural residents, we found that compared to urban residents, rural residents were more likely to be suffering from heart disease due to high parity. This difference was explained by the differences in living standards and medical and health conditions between urban and rural areas. In urban areas, better living standards can make up for the lack of nutrition, and better medical conditions can prevent the occurrence of chronic diseases. The double influence combined with living standards and medical conditions mediated the negative impact of multiple births. When adjusting the model only for women or men respectively, the association remained in the women group. The women experienced fertility behavior and undertook more family care, associated with a higher risk of heart disease than men.

Considering that the age at entry to parenthood was also associated with the risk of heart disease, late age at entry to parenthood was associated with a poorer risk level of heart disease. Our results were consistent with Lacey's study. The study of 7,600 British men and women found that giving birth before the age of 20 can cause women to be associated with an adverse cardiovascular profile in their middle age. On the contrary, parents who give birth at a later time have a lower vascular risk (21). The observed association between early age at entry to parenthood and most heart diseases (arrhythmia, angina pectoris, myocardial infarction, and heart failure) may reflect the direct biological effects of pregnancy and/or indirect effects through other metabolic and physiological changes (e.g., weight cycling) that increase the risk.

## Conclusions

On the whole, it can be considered that a similar association has been found in the elderly group of China. The high parity was associated with a high risk of heart disease and especially when the number of boys increased, the relationship was more obvious. When considering the difference in the region, the association only existed for the rural elderly and disappeared in urban. Besides, the late age at entry to parenthood associated with a poorer risk of heart disease was also found for the elderly in China. Based on our findings, some policy implications were being put forward. First, parents who were ready to give birth should be aware of the possible health loss of high parity. Postpartum nutrition supplement and chronic disease prevention were suggested to prevent heart disease in later life. Secondly, the elderly in rural areas should pay more attention to the heart disease. Participating in more daily exercise and physical examinations was a good choice to reduce the risk of heart disease. Thirdly, early age at entry to parenthood was not advocated. Based on our results, age at entry to parenthood was closely related to the risk of heart disease in later life. Especially, late age at entry to parenthood was associated with a lower risk of heart disease. Appropriately delaying the age at the first birth may help to prevent heart disease in later life. Meanwhile, the conclusions provide a lot of enlightenment of our fertility behavior and later-life health. From the stage of childbearing age, we should prevent the possible damage caused by childbirth, pay attention to nutrition supplements and recovery after birth, avoiding cumulative negative impact. From the perspective of old age, we should pay attention to physical health in old age. The disadvantage of physical health may increase the risk of heart disease. In addition, this paper has some limitations: firstly, the study does not discuss the endogeneity adequately, so the regression results may exist estimation biases; secondly, the research conclusions lack relevant medical explanations.

## Data Availability Statement

Publicly available datasets were analyzed in this study. This data can be found at: http://www.isss.pku.edu.cn/sjsj/charlsxm/index.htm.

## Author Contributions

YW has made a substantial contribution to the concept and design of the article in the writing and empirical analysis. HY, JP, and JWu collected data, analyzed data, and drafted the article. JWa, YY, YT, SC, LX, ShZ, Z-yL, ZS, and SiZ collected relevant literature and data, adjusted article format and carefully revised, and polished the language of article. All authors contributed to the article and approved the submitted version.

## Funding

This work was supported by the Later Funded Projects of National Social Science Foundation of China (grant number: 21FRKB003).

## Conflict of Interest

The authors declare that the research was conducted in the absence of any commercial or financial relationships that could be construed as a potential conflict of interest.

## Publisher's Note

All claims expressed in this article are solely those of the authors and do not necessarily represent those of their affiliated organizations, or those of the publisher, the editors and the reviewers. Any product that may be evaluated in this article, or claim that may be made by its manufacturer, is not guaranteed or endorsed by the publisher.

## Footnotes

^1^Data Source: The seventh National Census conducted in China.
